# Patterns of Genetic Diversity in Highly Invasive Species: Cogongrass (*Imperata cylindrica*) Expansion in the Invaded Range of the Southern United States (US)

**DOI:** 10.3390/plants9040423

**Published:** 2020-03-31

**Authors:** Rima D. Lucardi, Lisa E. Wallace, Gary N. Ervin

**Affiliations:** 1 USDA Forest Service, Southern Research Station, Forestry Sciences Laboratory, 320 East Green Street, Athens, GA 30602, USA; 2 Department of Biological Sciences, Old Dominion University, Norfolk, VA 23529, USA; 3 Department of Biological Sciences, Mississippi State University, Mississippi State, MS 39762, USA

**Keywords:** AFLP, genetic diversity, invasive, Poaceae, population genetics, range expansion

## Abstract

The spatial expansions of invasive organisms in the novel range are generally expected to follow an isolation-by-distance relationship (IBD) if the invasion is biologically driven; however, many invasions are facilitated anthropogenically. This research focused on the extant expansion patterns of cogongrass (*Imperata cylindrica*). Cogongrass is a widespread invasive species throughout the southern United States (US). Patterns of infestation vary among US states. Cogongrass is pyrogenic, and its invasion threatens softwood (*Pinus* spp.) plantations, a substantial economic market for this US region. Over 600 individuals were sampled from seven invaded US states, using amplified fragment length polymorphisms (AFLPs) to assess genetic diversity and population structure. We suspected that differences in historical management efforts among US states influenced differences in genetic diversity and structure. We detected two genetic lineages at the highest level of analysis. One genetic lineage was locally restricted, whereas the other was found throughout the study region. Admixed individuals were found in all US states and consistently co-occurred with the dominant lineage, suggesting that secondary contact and hybridization may have facilitated expansion. The widespread prevalence of only one of the two detected genetic lineages suggests a primary genetic lineage responsible for on-going population expansion in the US.

## 1. Introduction

Biological invasions continue to increase during this era of increased global connectivity [1], while research has sought to understand the biological mechanisms which contribute to novel invasion success or the failure to establish or expand beyond incipient populations [2]. Ultimately, the success of a novel plant invasion can be constrained by its biological and evolutionary limits, such as its evolutionary history, geographic origin, propagule pressure and multiple introductions, or a species’ ability to adapt to novel environments [3,4,5,6]. However, biology is not the only factor in successful plant invasions; the consequences of anthropogenic activity and vectoring are also influential [5,7,8]. It is known that changes in the structure and/or function of the recipient habitat may provide or limit opportunities for invasion success and can be possibly aided by human-assisted dispersal [9,10,11,12,13]. Disturbances can create new habitat resources (i.e., increased availability of canopy openings, or increased frequency of edge habitat) or facilitate the successful dispersal of invasive plant propagules (creation of “stepping-stone” patches, novel dispersal routes and/or mechanisms) in the spread and establishment of invasive plants [14,15,16]. Habitat-modifying activities by humans are common in the regular maintenance and management of rights-of-way, roads, and in agricultural and silvicultural practices in the United States (US). For many weedy plant species, human activities influence inter-patch connectivity by creating new habitats and by enhancing dispersal [17,18,19,20]. Long-distance dispersal can increase the overall rates of spread, but also has the potential to influence the population’s genetic structure by facilitating intraspecific hybridization among independent novel introductions or through the homogenization of a regional population of a particular plant invader.

Invasions, such as cogongrass (*Imperata cylindrica*), are of great concern to the agriculture of softwood timber plantations, primarily pines (*Pinus* spp.). Previous work on this species has shown that anthropogenic land use (e.g., the presence and maintenance of transportation corridors or forestry management practices) is an important driver of this species’ distribution, and therefore of range expansion [21,22]. Cogongrass (*Imperata cylindrica*) has invaded seven southern US states and has negatively impacted both the economy and the ecology of these states and the agronomy of the region [23,24]. This invasive grass is a federally listed noxious weed [25] and is considered a global weed of substantial consequence [26,27]. In addition to its invasion across the US softwood timber-growing region, cogongrass is widespread throughout the tropical and sub-tropical regions of the Old World [26,28]. In the US, this plant invasion has benefitted from multiple introductions from previously isolated genetic lineages [29,30,31]. Previous research observed two genetic lineages of parental material introduced from East Asia, along with documented occurrences of multiple introductions that probably facilitated the establishment of cogongrass in the southern US [31]. The continued spread of cogongrass into neighboring states is a significant concern [23,24,32]. Cogongrass is reported to be an obligate-outcrosser that produces thousands of viable seeds per inflorescence [23,33]. Despite its impressive sexual reproductive capacity, the range expansion of this species has been primarily attributed to the human-aided movement of rhizomatous fragments [24,30]. Again, previous research found cogongrass populations to be surprisingly genetically diverse for a clonally reproductive grass; therefore, this reproductive flexibility of both sexual and asexual approaches has contributed to its fitness and its successful establishment and invasion over the last 100 years [31]. Previous research also allows us to exclude interspecific hybridization with a congeneric directly contributing to current range expansion [34,35,36] at this time. Human-aided spread, both purposeful and inadvertent, is the probable vector of erratic range expansion, especially during the mid-20th century when propagules were transported from Mississippi and Alabama into Florida and elsewhere [29,30]. Consequences of cogongrass, in both the native and invaded ranges, include reductions in biodiversity, monotypic stands, and timber loss; furthermore, cogongrass is pyrogenic, meaning this grass species is highly-flammable, contributing to alterations in fire regimes to more frequent and/or intense fire events [23,24,26,33]. In managed timber plantations, young *Pinus* spp. are especially susceptible to cogongrass fire events; after a fire, cogongrass rhizomes are generally the first to re-sprout and then dominate the landscape, excluding other plants and animals from recolonizing [24,26,34].

Cogongrass was sampled from, and in cooperation with, state and federal agencies in the following US states experiencing extant infestation and practicing any form of current management: Alabama, Florida, Georgia, Louisiana, Mississippi, South Carolina and Texas. An anonymous genome-wide scan provided by amplified fragment length polymorphism (AFLPs) markers is considered adequate for this analysis. The same AFLP markers have been previously utilized to assess genetic diversity and population structure in invasive US cogongrass populations [31,36,37,38,39,40]; however, previous published research was limited in spatial scale. In this analysis, the US state-level genetic diversity and population structure across the infested range were evaluated. Furthermore, this evaluation sought if anthropogenic activities influenced the extant patterns of genetic diversity and population structure during novel range expansion. One example of a major anthropogenic influence is the American Recovery and Reinvestment Act (ARRA) of 2008, which allocated funding from the US federal government to state agencies for the purpose of improving the control and management of this invasive grass. Alabama received $6.3-mil (USD) and Georgia received $1.8-mil (USD) earmarked for cogongrass eradication. Mississippi received $1.2-mil (USD) and South Carolina received $700,000 (USD) for general invasive plant control and management. These widely variable financial allocations, and each state’s use of the funding, may have contributed to differential patterns of genetic diversity or structure among infested US states. Furthermore, we expected genetic variance among US states due to the historical treatment and management of cogongrass in duration, chemical or physical management, and historical efforts. Though state political borders are unlikely barriers to dispersal for propagule movement within the southern US, the influence of differential funding availability and state-level management practices on genetic diversity and structure are variable due to jurisdictional boundaries; thus, we expected cogongrass in states at the expanding fronts of the invasion (South Carolina and Texas) to be less genetically diverse than in the states that received direct introductions and where invasion has been present for the most amount of time (Mississippi and Alabama). Alternatively, states that received the most funding (e.g., Alabama and Georgia) may be less genetically diverse than states that received substantially less funding (e.g., South Carolina).

## 2. Results

### 2.1. Genetic Diversity

The AFLP analysis resulted in 2057 polymorphic loci from 676 cogongrass individuals. We observed a good reproducibility for this method (SE = 0.004; 95% CI; <1 mismatch per individual per locus) among the samples analyzed. The state-level average percentage of polymorphic loci was 23% (SE ± 7%), ranging from 2% (GA) to 56% (SC). The mean Shannon’s Information Index (I) was 0.030 (SE ± 0.001). Nei’s gene diversity for all states analyzed ranged from 0.006 (GA) to 0.042 (SC) with a mean of 0.023 (Table 1). Heterozygosity values also trended similarly to Shannon’s (I) and Nei’s gene diversity: the mean heterozygosity (both H_e_ and UH_e_) was 0.016 (SE ± 0.001). Spearman’s correlation coefficient (ρ) values between the sample size and genetic diversity were calculated for (1) all states, and (2) for states with more than 50 sampled individuals (AL, FL, LA, MS and SC). All relationships between the sample size and genetic diversity were not significant (*P* > 0.05), suggesting that unevenly sampling states did not unnecessarily bias results or interpretation.

**Figure 1:**
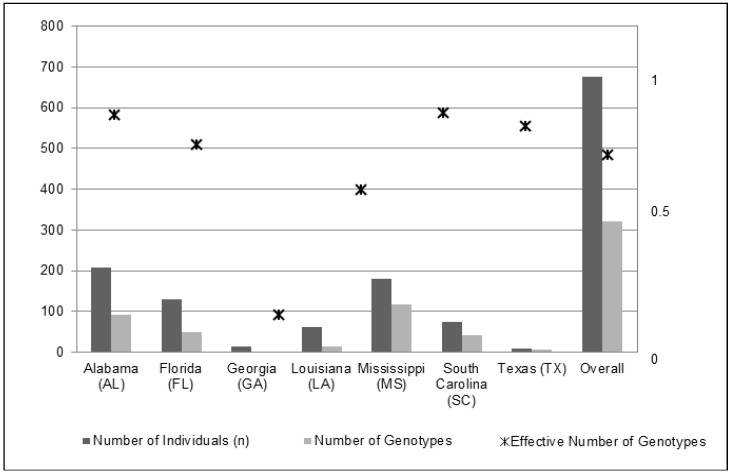
Genotypic diversity relative to the number of individual samples in each state and overall. The effective number of genotypes for the US state of Georgia was <1; therefore, no symbol is present for that state.

An overall reduction was observed in the clonal diversity analysis from 676 sampled individuals to 321 unique multi-locus genotypes (Table 1 and Figure 1). The mean genotype diversity (range: 0 to 1) for all states was 0.736 (SE ± 0.105). We observed the lowest genotype diversity in GA at 0.154, reducing the effective number of genotypes to 1.154. We observed the highest genotype diversity in MS and AL (>0.90), with FL and SC close behind (0.816 and 0.888, respectively; Table 1 and Figure 1).

### 2.2. Population Structure

**Figure 2:**
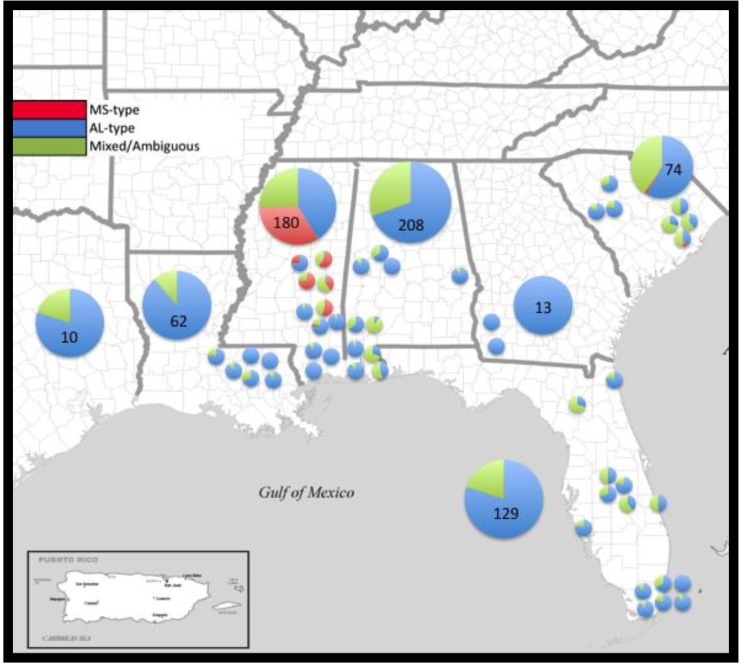
Map of the southern US with the proportion of individuals assigned to MS-type (red), AL-type (blue) or ambiguous (green) based on a 90% threshold of assignment from STRUCTURE.

**Figure 3:**
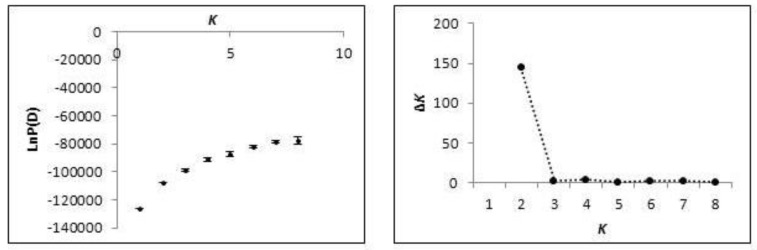
Summary evaluation of the posterior probability values [LnP(D)] used for the determination of the most likely number of clusters (*K*) from STRUCTURE simulations.

Of the 676 individuals sampled, 485 were assigned with >90% posterior probability to a single lineage; 73 to the MS-type lineage (red, Figure 1) and 412 to the AL-type lineage (blue, Figure 2). Admixture was present in both clusters (mean α = 0.165), and 191 individuals could not be confidently assigned to either cluster with strong confidence. A Bayesian cluster analysis was conducted in the program STRUCTURE, which supported an inference of two distinct genetic clusters (*K* = 2; mean LnP(D) = −107,561; Figure 3) extant in the region examined: a MS-type lineage and an AL-type lineage (Figure 2). Individuals assigned to the MS-type lineage are only present in central MS, based on our sampling, and there is a single outlier individual in SC. It is in this geographic locale of central MS that both lineages are present and co-occur at the patch level, with varying proportions of admixed individuals. In all other states in the region, the AL-type lineage is dominant and co-occurs with admixed individuals, except in GA where only the AL-type lineage was found. A cluster analysis at the patch- and state-level in GA is consistent with the genetic diversity, supporting a low heterogeneity, and all individuals in GA were assigned to the AL-type lineage. Additional Bayesian cluster analyses were conducted on all individuals excluding the individuals strongly assigned to the MS-type lineage. No population substructure was observed in the AL-type and in ambiguous individuals, and this remained consistent with the initial analysis.

Significant population pairwise F_ST_ values were observed between the two genetic groups within MS patches (F_ST_ = 0.330, *P* < 0.05; Table 2). Please note that the MS-central group was significantly dissimilar from all other groups tested (F_ST_ > 0.3); therefore, a pairwise F_ST_ analysis separated MS populations into two groups: 1) coastal Mississippi (MS-Coast) populations from the other 2) Mississippi lineage (MS-Central) (Table 2). The SC population was the least genetically differentiated from the FL populations (F_ST_ = 0.060), while the AL and SC populations were the least genetically differentiated from the TX population (F_ST_ = 0.094, 0.083, respectively; Table 2). Pairwise population F_ST_ values were similar between SC and most other states analyzed (F_ST_ < 0.2), excepting MS-central (F_ST_ = 0.314). Pairwise F_ST_ between TX and AL (F_ST_ = 0.090) was very similar to pairwise F_ST_ values between TX and SC, and between TX and FL (both F_ST_ = 0.083). The greatest genetic dissimilarity existed between TX and GA (F_ST_ = 0.553).

**Figure 4:**
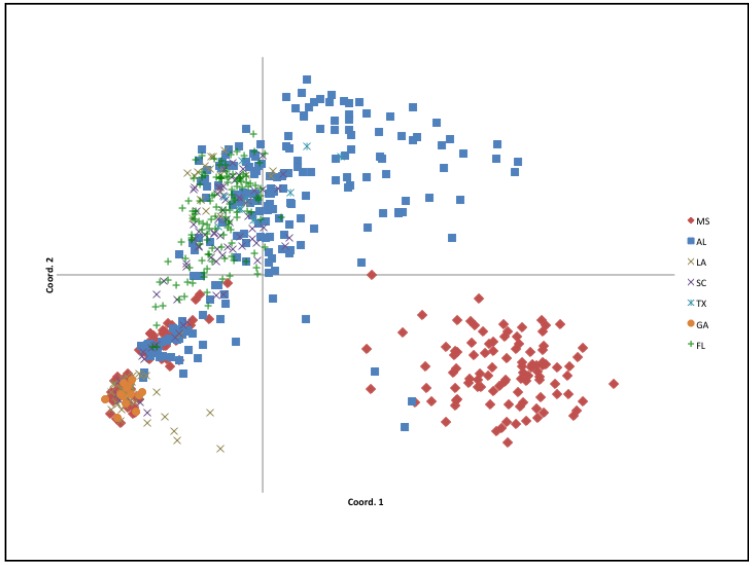
Principle coordinates analysis (PCoA) of individual genetic covariance with data standardization (N = 676, 61% of variation accounted for by the first two axes). All seven US states are represented in this ordination.

A principal coordinate analysis (PCoA) was consistent with the population structure as inferred by the Bayesian cluster analysis in STRUCTURE. The first two axes explained 61% of individual genetic variance (Figure 4). Two clusters were observed in the PCoA plot: one fairly contained cluster and the other being broad and loosely organized. Individuals sampled from MS were present in both clusters, where individuals sampled from central MS (MS-Central) appear localized to the bottom right quadrant (red diamonds, Figure 4) and individuals sampled from the MS Gulf Coast (MS-Coast) cluster with individuals from AL, FL and other states. Individuals sampled from AL are broadly distributed throughout the larger cluster, indicating a high degree of individual genetic variation. Individuals from FL also presented a similar pattern. Sampled individuals from TX (all collected from a single site) did not form a tight cluster, but grouped with samples from AL, FL, LA and SC. Georgia; these individuals from these states, however, form a very tight cluster within the broad cluster (Figure 4), overlapping within a subset of the AL, LA, MS and SC individuals, potentially indicating genetic relationships among geographically disparate populations.

The genetic differentiation was evaluated between the MS-type cluster and all other individuals based on inferences from STRUCTURE and the PCoA. Ambiguous individuals were lumped with the AL-type in an analysis of molecular variation (AMOVA; Table 3); this grouping was derived because of the low value of admixture from STRUCTURE (mean α = 0.165) and the high proportion of assignment to the AL-type in ambiguous individuals (<0.90, but >0.50). We observed a significant genetic differentiation (F_ST_ = 0.363, *P* < 0.001) between the groups as defined above.

## 3. Discussion

The detection of two distinct genetic lineages throughout the invaded region is consistent with other published research on the cogongrass invasion in the US [29,30,31,36]. This analysis, however, demonstrated that one of those two genetic lineages (the dominant AL-type) has been responsible for the majority of the spatial spread and persistence in the US. The dominant AL-type lineage is more heterogeneous, widespread, and is genetically distinct (F_ST_ = 0.363, *P* < 0.001) from the MS-type lineage, even when inclusive of ambiguously assigned individuals. A two-lineage scenario provided the only evidence of strong population structuring throughout the invaded US range. The majority of individuals sampled for this work were assigned with a 90% or greater probability to the AL-type lineage (Figure 2). The other detected genetic group (the MS-type) appears to be geographically constrained to central MS (Figure 2). This pattern may be due to the reduced competitive or dispersal ability of this lineage, lack of anthropogenic activity promoting its spread or other potentially heritable fitness factors restricting its ability to spread from this region.

The present analyses further demonstrated that, although the two genetic groups remain significantly differentiated at the scale of this study, admixture is present within individuals at the local, patch scale (mean α = 0.165). Admixture is also suggested to have increased upon regional evaluation as compared to previous research constrained to MS and AL (where mean α = 0.08) [31]. Similarly, recent admixture in the invasive weed, *Silene vulgaris*, was detected in the novel range (North America), and demonstrated a relationship between fitness benefits and increased heterozygosity in invasive populations [41]. Such results suggest the possibility that genetic mixing contributed to the successful spread of cogongrass across the southern US; however, measures of intraspecific phenotypic variation, and its relationship to genetic variation and/or hybridization, are needed to demonstrate whether significant genetic admixture has contributed to cogongrass’ success [42,43]. Typically, multiple introductions increase the genetic diversity and probability of success in a wide array of invasive, colonizing organisms [44,45,46,47,48].

Although we found a significant genetic distance to exist among the states (Table 2), the overwhelming evidence favored a regional population dominated by the AL-type lineage, with a highly-restricted MS-type found mainly restricted in the central portion of Mississippi (Figure 2). There also appears to be an element of biological influence on the population structure, as the majority of the genetic variation in cogongrass is partitioned within sampled locations (64%). Similar to previous findings at smaller scales (in terms of sample size and geography), the majority of genetic variation being partitioned within patches continues to be unexpected for a species thought to expand locally via clonal propagation. The regional degree of genetic differentiation in cogongrass (F_ST_ = 0.363, *P* < 0.001) is similar to that of early successional plant species (mean F_ST_ = 0.37) [49]. Even considering the fact that the majority of genetic differentiation is partitioned within local patches, average heterozygosity for state-level cogongrass sampling (H_e_ and UH_e_ = 0.016 ± 0.001) is still lower than such values in long-lived perennial plant species (H_e_ = 0.68) or those capable of selfing (H_e_ = 0.41) [49].

We expected that differential funding and historical management efforts among the seven US states would contribute to contemporary state-level genetic diversity differences across the region. Differing state-level management practices, efforts, and starting-acreages of infestation affected the relative genetic diversity (Table 1). The state of Alabama (AL) received the greatest allocation of cogongrass-specific funding from the American Recovery and Reinvestment Act (ARRA), $6.3-mil (USD). Despite receiving the largest allocation of federal funding, AL cogongrass populations on public and private lands were not much reduced since 2008. Though no published data has resulted since then, we do know that AL chose to outsource the ARRA funds to a private invasive plant control consulting firm responsible for the detection, treatment and repeated visits/re-application on infested sites. Since a follow-up chemical application is necessary in any cogongrass management plan or strategy [24], Alabama’s utilization of ARRA funds has been viewed as one of the greatest modern failures in cogongrass control efforts. Alabama continues to be one of the most infested states in the US, superseded only by Florida. When compared to the neighboring state of Georgia (GA), which received $1.8-mil (USD) from the ARRA specifically for cogongrass eradication, the state of GA resulted in the lowest genetic and genotypic diversity (Figure 2), along with the least amount of acreage infested excluding the infestation site in Texas. Additional evidence as to why GA is so different from other states in terms of the distribution and diversity of cogongrass infestation is that the Georgia Forestry Commission began treating cogongrass in the late 1960s (Art Miller personal correspondence, GFC *retired*) with chemical herbicide and mechanical removal decades before all other southern US states. Furthermore, the Georgia Forestry Commission currently utilizes a chemical-application strategy that has been most effective [24], along with semi-annual revisits to document infested sites. Therefore, the state of Georgia has been able to stem the majority of cogongrass infestation to the southwestern corner of the state, which borders Gulf Coastal Alabama. The states of GA and TX resulted in the lowest overall genetic diversity. We considered that the smaller sample sizes in these two states may have contributed to this pattern, 13 and 10 individuals, respectively; however, Spearman’s correlation tests did not find significant relationships between sample sizes and genetic/genotypic diversity outcomes (Figure 1, Table 1). The genotypic diversity in GA was slightly over 15%, whereas in TX it was over 77%. From our analysis, this suggested that cogongrass sites sampled in GA persist with both a low genetic and genotypic diversity. Furthermore, all individuals in GA were strongly assigned to the AL-type, also suggesting a superior ability to persist despite the fitness benefits expected to accompany a high genotypic diversity contributing to increased adaptability [3]. This pattern observed in GA, which is not mirrored in TX, indicates that the success of an invader can be based on the fitness of one to a few adapted genotypes [50,51].

Cogongrass possesses a broad global distribution [23,24], indicating a generalist phenotype, preferentially colonizing and positively associated with disturbance [21], and originating from potentially multiple points in Asia [29,30]. We consider cogongrass a suitable candidate for rapid adaptation and evolution in the invaded range due anthropogenic activities [52]. Furthermore, the chemical control of invasive plant populations is a human-induced selective pressure, unevenly applied and distributed across the landscape. These human activities directly and indirectly affect the population and genetic structure of secondarily invading populations, and will continue to do so.

The regional expansion of cogongrass since its initial introduction in 1919 has benefitted from the direct and purposeful anthropogenic transport of propagules, multiple introductions and secondary contact [29,30,31]. The majority of propagule spread had been previously attributed to the transport and establishment of rhizome fragments; however, the lack of a strong decay in isolation-by-distance (IBD) genetic-geographic relationships suggested this is not the case [31,36,40]. These data suggest that the cogongrass expansion throughout the South has probably benefitted from reproductive flexibility, the on-going movement of viable propagules (both seeds and rhizome fragments) and potential adaptive persistence under active management by chemical control. Given the clear regional dominance by the AL-type lineage of plants, further investigation into the mechanisms responsible for the successful invasion of cogongrass might benefit from a functional genetic comparison between the AL- and MS-lineage’s phenotypes and phenotypic responses, as well as intraspecific global references.

## 4. Materials and Methods

### 4.1. Study Area and Sampling

Live cogongrass leaf tissues were collected during 2008–2009 from seven states in the southern region of the US: Alabama (AL, n = 208, 10 patches), Florida (FL, n = 129, 14 patches), Georgia (GA, n = 13, 2 patches), Louisiana (LA, n = 62, 6 patches), Mississippi (MS, n = 180, 11 patches), South Carolina (SC, n = 74, 7 patches) and Texas (TX, n = 10, single patch site; Figure 2, Table 1, N = 676). Each tiller was assumed as being representative of an “individual” or a ramet in the patch/population (while acknowledging that individual patches may have arisen from only one to a few colonizing propagules resulting in few genets comprising a population). Leaf tissues were collected in the field from cogongrass populations. Patches were identified as contiguous sites of cogongrass, which often occur as circular patches in open areas or as long, narrow patches along roadside rights-of-way. Leaf tissues were placed into individually-labeled plastic bags and then stored in a cooler with 1–2 cups of ice (or ice substitute) per large cooler during collection and transport (for maximum 24–36 h, at ambient vehicle temperature). Silica gel containing color indicator was poured into individual bags to dry the leaf tissue in the lab upon unpacking of leaf tissues. Drying leaf tissues in silica gel were dried for at least 1 week at room temperature.

Cogongrass (*I. cylindrica*) is a listed Federal Noxious Weed; all sampling was conducted with approval by the U.S. Dept. of Agriculture, Animal and Plant Health Inspection Service, Plant Pest Quarantine (Permit#: P526P-12-00211, P526-080721-005). Additional permissions were required for access to specific areas, including a collection agreement with The Nature Conservancy (TNC) and Miami-Dade County Parks and Recreation (Permit #145). Most sampling was conducted on public land including National Forests (USDA Forest Service), interstate/highway rights-of-way and on private land with permission from state forestry agencies and associated landowners, as appropriate.

### 4.2. Molecular Analysis

Approximately 1-cm^2^ of dried individual leaf tissue was aseptically transferred into a 2-mL microcentrifuge tube for each individual. The extraction of total DNA from leaf tissues, primers, reagents and PCR conditions were described previously in Lucardi et al. [31,36] based on Vos et al. [53]. Lack of reproducibility is considered one of the negative aspects of dominant markers, such as AFLPs; therefore, we accompanied all runs with positive and negative controls.

Fragment data were visualized in GeneMarker^®^ (SoftGenetics, LLC, State College, PA, USA) and exported into a general text format. Detected fragments were sorted on migration size (basepairs) and objectively scored utilizing an independently developed procedure (Lucardi and Walker, unpublished methodology) using both Excel 2007 (Microsoft Corporation, Redmond, WA, USA) and PASW v.18.0 (SPSS, IBM Corporation, Armonk, NY, USA) as specified in Lucardi et al. [31,36].

Data conversions of presence-absence matrices were conducted in AFLPdat source script [54] in R v2.15.1. The genetic diversity was assessed using the expected heterozygosity (biased (H_e_) and unbiased (UH_e_)) and Shannon’s Information/Diversity Index (I), serving as a coefficient of similarity, for each state for state-level diversity estimates (GenAlEx 6.3) [55]. Population genetic diversity metrics, such as Shannon’s Information Index [I], were estimated from allele frequencies inferred from dominant data and are subject to Hardy-Weinberg equilibrium assumptions. These assumptions can reduce the accuracy in allele frequency estimations from dominant data, but reliable results for a comparative study can be achieved with adequate population sampling and a sufficient number of primer sets, which generate a large number of detected polymorphic loci [39,56,57].

Because of cogongrass’ capacity to clonally reproduce *via* rhizomes, we conducted a clonal analysis using the “Clones” function within the AFLPdat package [54]. The standard error among positive control replicates (0.004) functioned as the error parameter for detecting identical multi-locus genotypes. The number of unique multi-locus genotypes per population contributes toward a more accurate assessment of the actual genetic diversity. Uneven sample sizes may bias the interpretation of results due to statistical dependence between variables. We used the Spearman’s correlation coefficient to determine if relationships are present between sample size and genetic diversity. We tested the relationships between sample sizes and frequency-based estimates using Spearman’s correlation method (“cor.s”) in R v.2.15.1.

We evaluated the population structure with population pairwise F_ST_ (Arlequin v.3.5 [58]) and the Bayesian cluster analysis program, STRUCTURE v.2.3.3 [59], to infer the most likely number of clusters (or *K*) based on posterior log-likelihood probability values from each simulation. We utilized the Evanno et al. [60] method to detect the population structure when *K* ≥ 2. Multiple simulations were conducted for *K* = 1 through 7, based on the seven sampled US states, with the following parameters: admixture ancestry model (to infer α), burn-in 10,000 and 50,000 Markov Chain Monte Carlo (MCMC) [58]. The ad hoc statistic, Δ*K*, was plotted to determine the mode. Individuals were assigned to a single lineage if they contained a probability of membership of at least 0.90 (90% threshold) to one of the clusters; all other individuals that were not strongly assigned to one of the lineages were assigned as ambiguous. We further assessed the population structure with a principal coordinates analysis (PCoA, GenAlEx v.6.3) and analysis of molecular variation (AMOVA, [61]) performed in Arlequin v.3.5 [58] using genetic distances, testing the structure based on inferences made from multi-locus genetic information analyzed in a cluster analysis.

## Figures and Tables

**Table 1:** State-level sampling and location information with genetic and genotypic diversity estimates.

State	Counties Sampled	Other Location Information	Number of Individuals (n)	Shannon’s Information Index (I)	Nei’s Gene Diversity	Number of Genotypes	Effective Number of Genotypes
Alabama (AL)	Baldwin, Hale, Lee, Mobile, Washington, Sumter	Talladega NF, Frank Boykin WMA	208	0.033 ± 0.002	0.023	92	0.935
Florida (FL)	Alachua, Duval, Indian River, Miami-Dade, Osceola, Sarasota	Disney Wilderness Preserve (TNC), Miami-Dade municipal parks	129	0.033 ± 0.002	0.028	49	0.816
Georgia (GA)	Baker, Crawford, Decatur, Mitchell, Thomas, Worth	Georgia Forestry Commission	13	0.009 ± 0.001	0.006	2	0.154
Louisiana (LA)	St. Tammany, Washington	Benscreek WMA	62	0.024 ± 0.002	0.029	14	0.64
Mississippi (MS)	Greene, Harrison, Jasper, Jones, Scott, Smith, Wayne	Desoto NF, Bienville NF	180	0.038 ± 0.003	0.023	117	0.94
South Carolina (SC)	Berkeley, Greenwood, Saluda, Union	Frances Marion NF, Sumter NF	74	0.063 ± 0.002	0.042	41	0.888
Texas (TX)	Tyler	Texas Forest Service	10	0.012 ± 0.002	0.011	6	0.778
Overall			676			321	
Mean (±SE)				0.030 ± 0.001	0.023 ± 0.001		0.736 ± 0.105

**Table 2:** Seven groups analyzed for population pairwise F_ST_^1^, where MS-type (central) was separated from coast (AL-type) for the state-level analysis.

MS-Coast	AL	LA	SC	TX	GA	FL	
**0.132**	*						**AL**
**0.096**	**0.175**	*					**LA**
**0.098**	**0.100**	**0.120**	*				**SC**
**0.217**	**0.090**	**0.264**	**0.083**	*			**TX**
**0.150**	**0.277**	**0.050**	**0.158**	**0.553**	*		**GA**
**0.146**	**0.094**	**0.158**	**0.060**	**0.083**	**0.281**	*	**FL**
**0.330**	**0.320**	**0.444**	**0.314**	**0.348**	**0.478**	**0.385**	**MS-Central**

^1^ Significant values in bold, *P* = 0.05; * indicates a value of 0, when pairwise comparison of the same group.

**Table 3:** Analysis of molecular variance (AMOVA) where two groups were inferred from the STRUCTURE analysis.

Source of Variation	d.f.	Sum of Squares	Percentage of Variation	*P*-Value
Among Groups	1	1325.57	27.43	<0.001
Among populations within groups	6	1283.18	8.88	0.110
Within populations	668	12,151.01	63.68	<0.001
Total	675	14,776.76		
F_ST_ = 0.363 (*P* < 0.001), F_SC_ = 0.122 (*P* < 0.001), F_CT_ = 0.274 (*P* = 0.11)				

## Data Accessibility

Sampling coordinates are detailed in the Appendix section of Lucardi 2012, available freely here: [http://sun.library.msstate.edu/ETD-db/theses/available/etd-10192012-122638/unrestricted/LUCARDI_Lib_v2.3_291012.pdf].
